# Trends in Sugar-Sweetened Beverage Intake Among Young Children, United States, 2021–2023

**DOI:** 10.5888/pcd22.250195

**Published:** 2025-09-04

**Authors:** Mary Ellen Grap, Sofia Awan, Carrie A. Dooyema, Julie L. Self, Ann M. Goding Sauer, Kristin J. Marks, Heather C. Hamner

**Affiliations:** 1 Division of Nutrition, Physical Activity, and Obesity, National Center for Chronic Disease Prevention and Health Promotion, Centers for Disease Control and Prevention, Atlanta, Georgia; 2 Oak Ridge Institute for Science and Education, Oak Ridge, Tennessee; 3 US Public Health Service Commissioned Corps, Rockville, Maryland

## Abstract

We used data from the National Survey of Children’s Health in 2021, 2022, and 2023 to examine trends in sugar-sweetened beverage (SSB) intake among children aged 1 to 5 years in the US. We performed trend analyses nationally, by age group, and by state and jurisdiction (District of Columbia) for 2 frequencies of intake: 1 to 3 times per week and 4 or more times per week, with “none” as the referent group. We found no significant linear trends at the national level. Two states had significant linear trends for SSB intake 1 to 3 times per week, and 6 states and the District of Columbia had significant linear trends for SSB intake ≥4 times per week. State public health professionals can use these data to understand recent SSB intake among children.

**Summary**

**What is already known on this topic?**

Sugar-sweetened beverages (SSBs) are a leading source of added sugars in the diets of young children in the US, and many children exceed recommendations for added sugars.

**What is added by this report?**

National estimates from the National Survey of Children’s Health (2021–2023) show that SSB intake among young children has remained largely stable, although intake decreased in a few states.

**What are the implications for public health practice?**

Emphasizing the importance of limiting intake of added sugars in existing state or jurisdictional and national programs and policies that affect young children could improve their nutrition and support optimal growth and health. Understanding progress in some states or jurisdictions may be important for designing public health programs.

## Objective

Nutrition during the first 5 years of life is critical to support the health and development of children (1). Consumption of added sugars is associated with type 2 diabetes, metabolic syndrome, cardiovascular disease, obesity, asthma, and dental caries (2,3). Sugar-sweetened beverages (SSBs) are the leading contributor of added sugars to the diets of young children in the US (4). The *Dietary Guidelines for Americans 2020–2025 *recommends that children younger than 2 years do not consume any added sugars and children 2 years or older limit added sugars to less than 10% of daily calories (1). The National Survey of Children’s Health (NSCH) first collected data on SSB intake among young children in 2021. It found that 36.4% of children aged 1 to 5 years consumed SSBs 1 to 3 times in the previous week, 21.0% consumed SSBs 4 or more times per week, and prevalence varied by state (5,6). A gap exists in understanding how the prevalence of SSB consumption has changed nationally and by state and jurisdiction since 2021. A previous study found a significant decrease in SSB intake among children aged 2 to 8 years from 2001 to 2018 (4). To understand more recent trends, we examined SSB intake nationally and by the 50 states and 1 jurisdiction (District of Columbia) among children aged 1 to 5 years from 2021 through 2023.

## Methods

NSCH is a nationally representative survey that collects data on the physical and emotional health of noninstitutionalized children aged 0 to 17 years in the US (7). The survey is funded and directed by the Health Resources and Services Administration’s Maternal and Child Health Bureau and is conducted by the US Census Bureau; data have been collected annually since 2016 for approximately 6 or 7 months from June or July to mid-January of the next year (7). Households are randomly sampled, and 1 child or adolescent is selected. Age-specific questionnaires are completed by an adult familiar with the selected child’s or adolescent’s health and health care (7). 

We assessed changes in SSB consumption nationally and by state and jurisdiction over time among children aged 1 to 5 years by using data from 3 survey years: 2021 (N = 18,551), 2022 (N = 17,534), and 2023 (N = 19,314). The weighted overall response rates for NSCH were 40.3% in 2021, 39.1% in 2022, and 35.8% in 2023 (7–9). Beginning in 2021, caregivers were asked, “During the past week, how many times did this child drink sugary drinks such as soda, fruit drinks, sports drinks, or sweet tea? (Do not include 100% fruit juice.)” (7). Response options were categorized into 3 frequency groups: none, 1 to 3 times per week, or 4 or more times per week.

We calculated weighted percentages and 95% CIs for SSB intake nationally according to NSCH guidance, nationally by age group (aged 1–2 and 3–5 years) and by state or jurisdiction. We used SAS-callable SUDAAN version 11 (RTI International) to conduct a trend analysis. We used polynomial logistic regression with a quadratic time variable to test nonlinearity. Where we found no evidence of nonlinearity, we ran a logistic regression model with only a linear time variable. The reference group was children who had no intake of SSBs (“none”). We considered *P* values of <.05 significant. This activity was reviewed by the Centers for Disease Control and Prevention, deemed research not involving human subjects, and was conducted consistent with applicable federal law and CDC policy.

## Results

**Figure 1:**
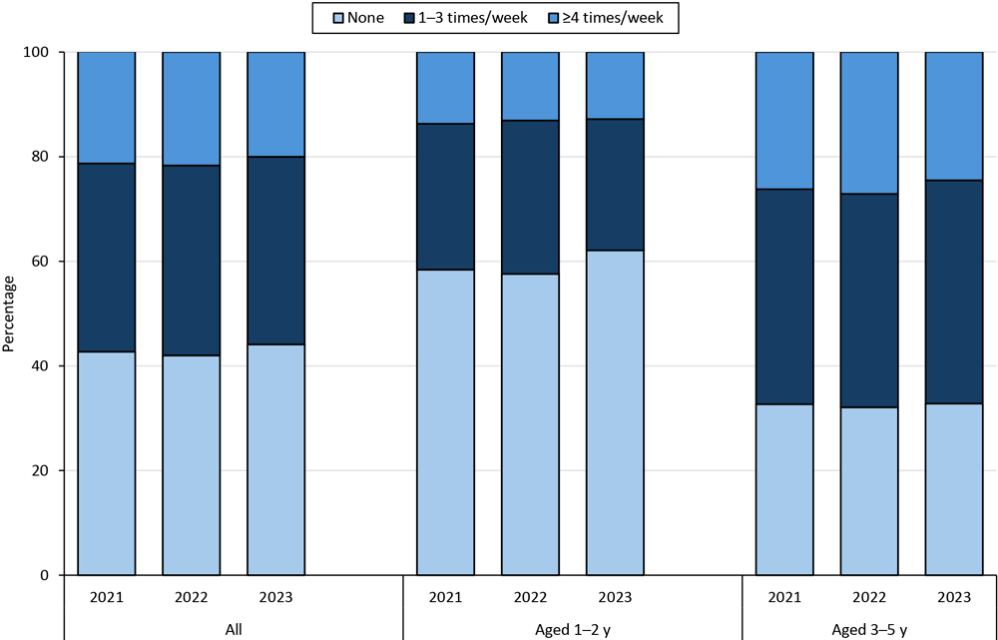
National prevalence of sugar-sweetened beverage intake, by frequency and survey year, among all children aged 1 to 5 years and by age group, National Survey of Children’s Health 2021–2023.

Nationally, among children aged 1 to 5 years, the prevalence of no SSB intake in the past week was 42.7% in 2021, 42.0% in 2022, and 44.1% in 2023. We found no significant trend in the prevalence of young children who consumed SSBs 1 to 3 times per week (2021, 36.0%; 2022, 36.3%; 2023, 35.9%; *P* = .52). The prevalence of SSB intake 4 or more times per week was also stable during the study period (2021, 21.4%; 2022, 21.7%; 2023, 20.0%; *P* = .17) (Figure 1, Appendix Table 1). Children aged 3 to 5 years had a greater frequency of SSB intake overall when compared with children aged 1 to 2 years for all years (2021–2023).

**Table:** 

Age group, y	None			1–3 Times per week			≥4 Times per week		
	2021	2022	2023	2021	2022	2023	2021	2022	2023
All	42.7	42.0	44.1	36.0	36.3	35.9	21.4	21.7	20.0
1–2	58.4	57.6	62.1	27.9	29.3	25.1	13.7	13.1	12.8
3–5	32.7	32.1	32.8	41.1	40.8	42.7	26.2	27.1	24.5

**Figure 2:**
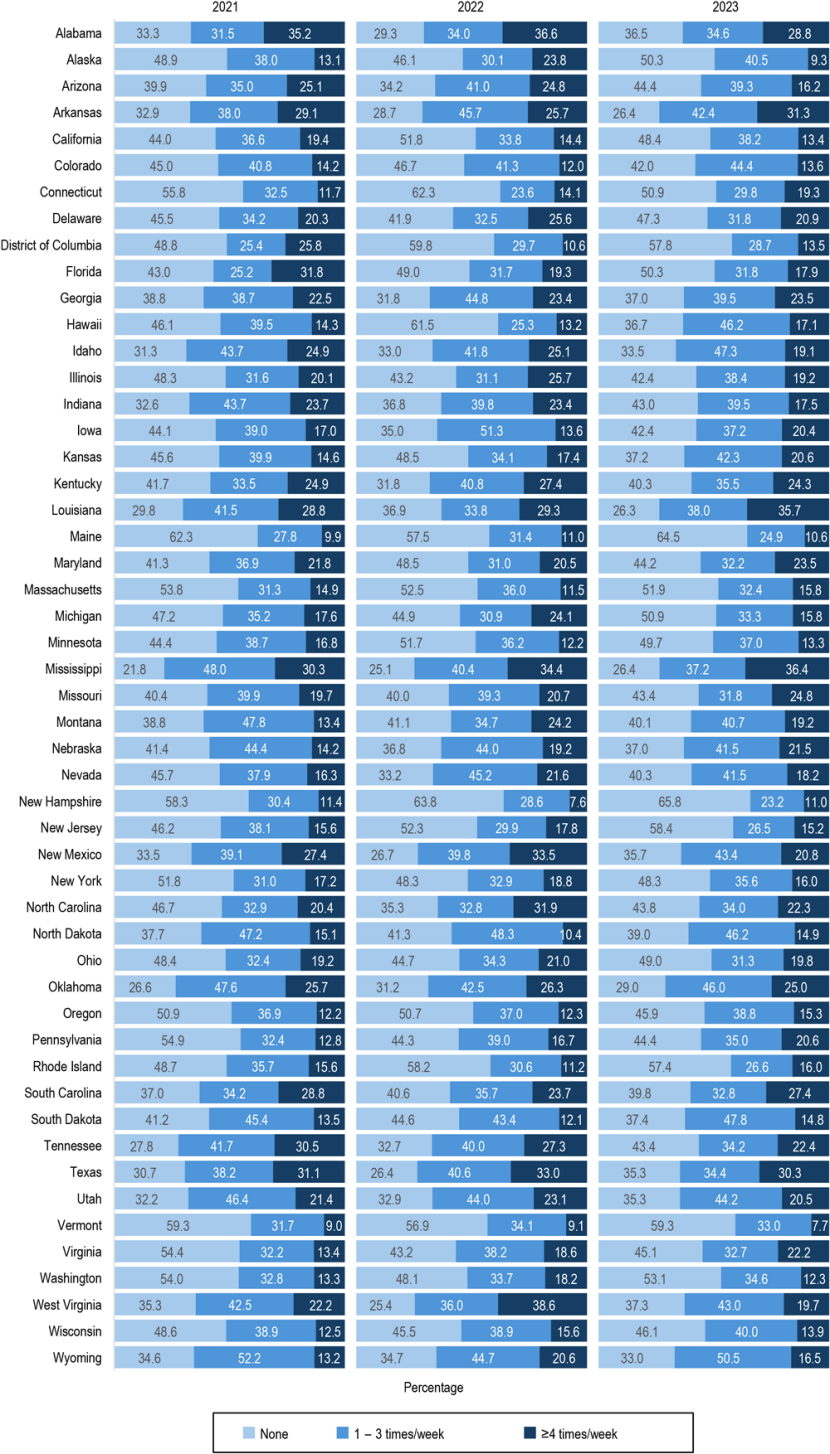
Trends in sugar-sweetened beverage (SSB) intake among children aged 1 to 5 years, by frequency and state, National Survey of Children’s Health 2021–2023. A significant decreasing linear trend in SSB intake 1–3 times per week was found in New Jersey and Tennessee. A significant linear trend for SSB intake ≥4 times per week was found in Kansas, Pennsylvania, and Virginia (increasing) and in the District of Columbia, Florida, Indiana, and Tennessee (decreasing).

We found differences in SSB intake at the state and jurisdictional levels. In 2023, the prevalence of children aged 1 to 5 years who reported no SSB intake in the past week ranged from 26.3% in Louisiana to 65.8% in New Hampshire (Figure 2, Appendix Table 2). In that year, SSB intake 1 to 3 times per week ranged from 23.2% in New Hampshire to 50.5% in Wyoming, and SSB intake 4 or more times per week ranged from 7.7% in Vermont to 36.4% in Mississippi. Two states, New Jersey and Tennessee, had significant decreasing linear trends for SSB intake 1 to 3 times per week. Three states — Florida, Indiana, and Tennessee — and the District of Columbia had significant decreasing linear trends for SSB intake 4 or more times per week when compared with no SSB intake in the past week. Three states — Kansas, Pennsylvania, and Virginia — had significant increasing linear trends for SSB intake 4 or more times per week.

**Table:** 

State	2021			2022			2023		
	None	1–3 Times per week	≥4 Times per week	None	1–3 Times per week	≥4 Times per week	None	1–3 Times per week	≥4 Times per week
Alabama	33.3	31.5	35.2	29.3	34.0	36.6	36.5	34.6	28.8
Alaska	48.9	38.0	13.1	46.1	30.1	23.8	50.3	40.5	9.3
Arizona	39.9	35.0	25.1	34.2	41.0	24.8	44.4	39.3	16.2
Arkansas	32.9	38.0	29.1	28.7	45.7	25.7	26.4	42.4	31.3
California	44.0	36.6	19.4	51.8	33.8	14.4	48.4	38.2	13.4
Colorado	45.0	40.8	14.2	46.7	41.3	12.0	42.0	44.4	13.6
Connecticut	55.8	32.5	11.7	62.3	23.6	14.1	50.9	29.8	19.3
Delaware	45.5	34.2	20.3	41.9	32.5	25.6	47.3	31.8	20.9
District of Columbia	48.8	25.4	25.8	59.8	29.7	10.6	57.8	28.7	13.5
Florida	43.0	25.2	31.8	49.0	31.7	19.3	50.3	31.8	17.9
Georgia	38.8	38.7	22.5	31.8	44.8	23.4	37.0	39.5	23.5
Hawaii	46.1	39.5	14.3	61.5	25.3	13.2	36.7	46.2	17.1
Idaho	31.3	43.7	24.9	33.0	41.8	25.1	33.5	47.3	19.1
Illinois	48.3	31.6	20.1	43.2	31.1	25.7	42.4	38.4	19.2
Indiana	32.6	43.7	23.7	36.8	39.8	23.4	43.0	39.5	17.5
Iowa	44.1	39.0	17.0	35.0	51.3	13.6	42.4	37.2	20.4
Kansas	45.6	39.9	14.6	48.5	34.1	17.4	37.2	42.3	20.6
Kentucky	41.7	33.5	24.9	31.8	40.8	27.4	40.3	35.5	24.3
Louisiana	29.8	41.5	28.8	36.9	33.8	29.3	26.3	38.0	35.7
Maine	62.3	27.8	9.9	57.5	31.4	11.0	64.5	24.9	10.6
Maryland	41.3	36.9	21.8	48.5	31.0	20.5	44.2	32.2	23.5
Massachusetts	53.8	31.3	14.9	52.5	36.0	11.5	51.9	32.4	15.8
Michigan	47.2	35.2	17.6	44.9	30.9	24.1	50.9	33.3	15.8
Minnesota	44.4	38.7	16.8	51.7	36.2	12.2	49.7	37.0	13.3
Mississippi	21.8	48.0	30.3	25.1	40.4	34.4	26.4	37.2	36.4
Missouri	40.4	39.9	19.7	40.0	39.3	20.7	43.4	31.8	24.8
Montana	38.8	47.8	13.4	41.1	34.7	24.2	40.1	40.7	19.2
Nebraska	41.4	44.4	14.2	36.8	44.0	19.2	37.0	41.5	21.5
Nevada	45.7	37.9	16.3	33.2	45.2	21.6	40.3	41.5	18.2
New Jersey	46.2	38.1	15.6	52.3	29.9	17.8	58.4	26.5	15.2
New Mexico	33.5	39.1	27.4	26.7	39.8	33.5	35.7	43.4	20.8
New York	51.8	31.0	17.2	48.3	32.9	18.8	48.3	35.6	16.0
New Hampshire	58.3	30.4	11.4	63.8	28.6	7.6	65.8	23.2	11.0
North Carolina	46.7	32.9	20.4	35.3	32.8	31.9	43.8	34.0	22.3
North Dakota	37.7	47.2	15.1	41.3	48.3	10.4	39.0	46.2	14.9
Ohio	48.4	32.4	19.2	44.7	34.3	21.0	49.0	31.3	19.8
Oklahoma	26.6	47.6	25.7	31.2	42.5	26.3	29.0	46.0	25.0
Oregon	50.9	36.9	12.2	50.7	37.0	12.3	45.9	38.8	15.3
Pennsylvania	54.9	32.4	12.8	44.3	39.0	16.7	44.4	35.0	20.6
Rhode Island	48.7	35.7	15.6	58.2	30.6	11.2	57.4	26.6	16.0
South Carolina	37.0	34.2	28.8	40.6	35.7	23.7	39.8	32.8	27.4
South Dakota	41.2	45.4	13.5	44.6	43.4	12.1	37.4	47.8	14.8
Tennessee	27.8	41.7	30.5	32.7	40.0	27.3	43.4	34.2	22.4
Texas	30.7	38.2	31.1	26.4	40.6	33.0	35.3	34.4	30.3
Utah	32.2	46.4	21.4	32.9	44.0	23.1	35.3	44.2	20.5
Vermont	59.3	31.7	9.0	56.9	34.1	9.1	59.3	33.0	7.7
Virginia	54.4	32.2	13.4	43.2	38.2	18.6	45.1	32.7	22.2
Washington	54.0	32.8	13.3	48.1	33.7	18.2	53.1	34.6	12.3
West Virginia	35.3	42.5	22.2	25.4	36.0	38.6	37.3	43.0	19.7
Wisconsin	48.6	38.9	12.5	45.5	38.9	15.6	46.1	40.0	13.9
Wyoming	34.6	52.2	13.2	34.7	44.7	20.6	33.0	50.5	16.5

## Discussion

We investigated trends of SSB intake among children aged 1 to 5 years in the US at the national and state or jurisdictional level using data from 2021 through 2023. We found that SSB intake was stable at the national level and among age groups during the 3 years examined. However, we observed several significant trends among states and the District of Columbia.

Although we found a significant increase in SSB intake in 3 states from 2021 to 2023, SSB intake significantly decreased in 4 states and the District of Columbia. Tennessee had significant decreasing trends in both frequencies of SSB intake: 1 to 3 times per week and 4 or more times per week. Factors affecting SSB intake may be multifaceted; therefore, trends may not be explained by a single, specific change but may be affected by factors at the individual, household, environmental, and policy levels (5,10). Multilevel approaches could address SSB intake among children. States and jurisdictions could use various approaches to reduce intake, such as early care and education licensing to prohibit serving SSBs, as was enacted in Tennessee in 2022 (11), and educational campaigns at the state and jurisdictional levels, such as Rethink Your Drink in California (12) and NJSugarfreed in New Jersey (12).

This study has several strengths. The data are timely and nationally representative. In addition, because of oversampling, the study provides representative estimates at the state and jurisdictional level for children aged 1 to 5 years. This is the first time that SSB trends for this age group could be analyzed at the state and jurisdictional levels. The study’s limitations include potential recall and social desirability bias of the caregiver report. NSCH did not collect data on the type and quantity of SSBs consumed, which limits interpretability and direct comparisons with recommendations of the *Dietary Guidelines for Americans 2020–2025*. NSCH also did not collect information to explain why intake changed in some states. In addition, only 3 years of SSB data with oversampling of this age group are currently available. Continued surveillance is important to understand long-term trends of SSB intake among young children.

Nationally, SSB intake among young children was stable during the study period, while some states and the District of Columbia saw significant decreases in intake. Understanding actions taken by these states and this jurisdiction that might be contributing to reduced SSB intake is important to help other states and jurisdictions apply strategies to support optimal nutrition among young children in the US.

## Appendix Supplemental Tables

**Appendix Table 1:** National Prevalence, Including 95% CIs, of Sugar-Sweetened Beverage Intake, by Frequency and Survey Year, Among All Children Aged 1 to 5 Years and by Age Group, National Survey of Children’s Health 2021–2023

Age group, y	None, % (95% CI)			1-3 Times per week, % (95% CI)				≥4 Times per week, % (95% CI)			
	2021	2022	2023	2021	2022	2023	*P* value^a^	2021	2022	2023	*P* value^b^
All	42.7 (41.0–44.3)	42.0 (40.5–43.5)	44.1 (42.6–45.6)	36.0 (34.3–37.6)	36.3 (34.9–37.8)	35.9 (34.5–37.4)	.52	21.4 (19.8–23.0)	21.7 (20.3–23.2)	20.0 (18.6–21.5)	.17
1–2	58.4 (55.6–61.2)	57.6 (55.0–60.3)	62.1 (59.3–64.8)	27.9 (25.3–30.6)	29.3 (26.8–31.8)	25.1 (22.9–27.4)	.08	13.7 (11.9–15.7)	13.1 (11.4–15.1)	12.8 (10.6–15.4)	.35
3–5	32.7 (30.8–34.7)	32.1 (30.5–33.9)	32.8 (31.2–34.5)	41.1 (38.9–43.2)	40.8 (38.9–42.6)	42.7 (40.8–44.5)	.56	26.2 (24.0–28.5)	27.1 (25.2–29.1)	24.5 (22.8–26.4)	.42

^a^ *P* value testing for linear trend for 1–3 times per week from logistic regression modeling with a linear time variable and “none” as the referent group.

^b^ *P* value testing for linear trend for ≥4 times per week from logistic regression modeling with a linear time variable and “none” as the referent group.

**Appendix Table 2:** Trends in Sugar-Sweetened Beverage (SSB) Intake Among Children Aged 1 to 5 Years, by Frequency and State, Including 95% CIs and Tests for Trend, National Survey of Children’s Health 2021–2023

State or jurisdiction	None, % (95% CI)			1–3 Times per week, % (95% CI)			≥4 Times per week, % (95% CI)		
	2021	2022	2023	2021	2022	2023	2021	2022	2023
All, n	8,700	8,121	9,142	6,742	6,371	7,106	3,109	3,042	3,066
Alabama	33.3 (26.3–41.2)	29.3 (22.5–37.2)	36.5 (29.4–44.3)	31.5 (25.5–38.1)	34.0 (26.5–42.5)	34.6 (28.0–42.0)	35.2 (28.0–43.1)	36.6 (28.2–46.0)	28.8 (22.2–36.4)
Alaska^a^	48.9 (40.4–57.4)	46.1 (37.6–54.9)	50.3 (41.2–59.3)	38.0 (30.1–46.6)	30.1 (22.5–38.9)	40.5 (31.9–49.7)	13.1 (8.2–20.5)	23.8 (16.4–33.3)	9.3 (5.1–16.3)
Arizona	39.9 (31.7–48.7)	34.2 (25.9–43.6)	44.4 (35.6–53.6)	35.0 (27.6–43.3)	41.0 (31.8–51.0)	39.3 (31.3–48.0)	25.1 (17.3–34.9)	24.8 (16.6–35.3)	16.2 (11.3–22.9)
Arkansas	32.9 (25.6–41.1)	28.7 (21.9–36.6)	26.4 (20.7–33.0)	38.0 (30.0–46.7)	45.7 (37.3–54.3)	42.4 (34.8–50.3)	29.1 (22.0–37.5)	25.7 (19.4–33.1)	31.3 (23.8–39.9)
California	44.0 (36.8–51.5)	51.8 (46.7–56.8)	48.4 (44.6–52.3)	36.6 (29.1–44.7)	33.8 (29.3–38.7)	38.2 (34.4–42.1)	19.4 (13.5–27.2)	14.4 (11.2–18.4)	13.4 (10.9–16.4)
Colorado	45.0 (38.9–51.3)	46.7 (40.0–53.5)	42.0 (35.6–48.7)	40.8 (34.7–47.2)	41.3 (34.8–48.2)	44.4 (37.7–51.3)	14.2 (9.9–19.9)	12.0 (8.3–17.1)	13.6 (9.1–19.9)
Connecticut	55.8 (47.6–63.8)	62.3 (54.5–69.5)	50.9 (42.3–59.5)	32.5 (25.0–40.9)	23.6 (18.4–29.7)	29.8 (22.7–37.9)	11.7 (7.0–19.0)	14.1 (8.7–21.9)	19.3 (13.0–27.6)
Delaware	45.5 (37.8–53.5)	41.9 (33.2–51.2)	47.3 (38.7–55.9)	34.2 (26.8–42.4)	32.5 (24.3–41.9)	31.8 (24.7–39.8)	20.3 (14.1–28.2)	25.6 (18.0–35.0)	20.9 (14.1–30.0)
District of Columbia^b^	48.8 (39.5–58.3)	59.8 (50.2–68.6)	57.8 (48.0–67.0)	25.4 (17.7–35.0)	29.7 (21.7–39.2)	28.7 (20.4–38.7)	25.8 (17.6–36.2)	10.6 (5.6–18.9)	13.5 (7.7–22.8)
Florida^c^	43.0 (34.9–51.5)	49.0 (40.4–57.7)	50.3 (41.9–58.7)	25.2 (19.0–32.6)	31.7 (24.5–39.8)	31.8 (24.6–40.0)	31.8 (23.7–41.1)	19.3 (13.0–27.8)	17.9 (11.7–26.4)
Georgia	38.8 (32.7–45.3)	31.8 (25.9–38.4)	37.0 (30.2–44.3)	38.7 (32.5–45.3)	44.8 (37.6–52.1)	39.5 (32.2–47.3)	22.5 (17.2–28.9)	23.4 (17.9–30.0)	23.5 (17.3–31.1)
Hawaii^d^	46.1 (39.0–53.4)	61.5 (52.1–70.1)	36.7 (28.3–45.9)	39.5 (32.4–47.1)	25.3 (18.3–33.9)	46.2 (36.9–55.8)	14.3 (10.4–19.4)	13.2 (7.8–21.5)	17.1 (10.6–26.5)
Idaho	31.3 (24.4–39.3)	33.0 (26.2–40.6)	33.5 (26.0–42.0)	43.7 (35.5–52.4)	41.8 (34.1–50.0)	47.3 (38.4–56.4)	24.9 (18.2–33.1)	25.1 (18.5–33.2)	19.1 (13.3–26.7)
Illinois	48.3 (40.9–55.8)	43.2 (35.5–51.3)	42.4 (36.7–48.3)	31.6 (25.5–38.4)	31.1 (24.7–38.3)	38.4 (32.7–44.3)	20.1 (14.1–27.9)	25.7 (18.7–34.1)	19.2 (14.6–24.9)
Indiana^e^	32.6 (26.6–39.4)	36.8 (29.9–44.3)	43.0 (35.9–50.4)	43.7 (36.3–51.3)	39.8 (32.4–47.7)	39.5 (32.2–47.2)	23.7 (17.5–31.2)	23.4 (17.1–31.0)	17.5 (12.7–23.6)
Iowa^f^	44.1 (36.9–51.5)	35.0 (28.2–42.5)	42.4 (34.6–50.6)	39.0 (32.1–46.3)	51.3 (43.2–59.4)	37.2 (29.7–45.4)	17.0 (12.4–22.8)	13.6 (9.6–19.0)	20.4 (13.9–29.0)
Kansas^g^	45.6 (38.6–52.7)	48.5 (40.1–57.1)	37.2 (33.0–41.5)	39.9 (33.3–46.7)	34.1 (26.9–42.0)	42.3 (37.8–46.9)	14.6 (10.2–20.4)	17.4 (11.8–24.9)	20.6 (16.9–24.9)
Kentucky	41.7 (34.0–49.8)	31.8 (25.0–39.5)	40.3 (32.5–48.6)	33.5 (26.8–40.9)	40.8 (32.7–49.4)	35.5 (27.9–43.9)	24.9 (19.0–31.8)	27.4 (20.4–35.8)	24.3 (18.2–31.6)
Louisiana	29.8 (23.0–37.6)	36.9 (28.5–46.2)	26.3 (22.3–30.8)	41.5 (33.9–49.4)	33.8 (26.1–42.6)	38.0 (33.1–43.2)	28.8 (22.0–36.6)	29.3 (21.6–38.4)	35.7 (30.6–41.1)
Maine	62.3 (55.6–68.5)	57.5 (49.9–64.8)	64.5 (56.4–71.8)	27.8 (22.3–34.1)	31.4 (24.8–39.0)	24.9 (18.7–32.3)	9.9 (6.9–14.0)	11.0 (7.3–16.3)	10.6 (6.5–16.9)
Maryland	41.3 (33.0–50.2)	48.5 (40.0–57.0)	44.2 (36.2–52.6)	36.9 (28.8–45.9)	31.0 (23.9–39.2)	32.2 (25.0–40.4)	21.8 (14.8–30.7)	20.5 (13.9–29.1)	23.5 (16.5–32.4)
Massachusetts	53.8 (45.4–62.0)	52.5 (44.0–60.9)	51.9 (43.3–60.3)	31.3 (24.2–39.4)	36.0 (27.9–44.8)	32.4 (25.2–40.5)	14.9 (9.2–23.3)	11.5 (6.6–19.3)	15.8 (9.3–25.4)
Michigan	47.2 (39.4–55.1)	44.9 (37.2–52.9)	50.9 (43.3–58.5)	35.2 (28.1–43.0)	30.9 (24.6–38.1)	33.3 (26.6–40.8)	17.6 (12.7–23.9)	24.1 (17.4–32.5)	15.8 (10.7–22.7)
Minnesota	44.4 (36.6–52.6)	51.7 (44.0–59.3)	49.7 (44.8–54.5)	38.7 (30.7–47.4)	36.2 (29.3–43.7)	37.0 (32.4–41.8)	16.8 (10.8–25.2)	12.2 (7.4–19.3)	13.3 (9.9–17.8)
Mississippi	21.8 (16.1–28.8)	25.1 (17.4–34.9)	26.4 (19.9–34.1)	48.0 (39.7–56.3)	40.4 (31.7–49.8)	37.2 (28.9–46.4)	30.3 (23.4–38.1)	34.4 (26.0–44.0)	36.4 (28.0–45.8)
Missouri	40.4 (32.8–48.4)	40.0 (32.1–48.5)	43.4 (35.6–51.5)	39.9 (32.4–47.8)	39.3 (31.4–47.8)	31.8 (25.3–39.0)	19.7 (14.0–27.1)	20.7 (14.5–28.6)	24.8 (17.3–34.4)
Montana	38.8 (31.1–47.2)	41.1 (33.2–49.5)	40.1 (32.4–48.4)	47.8 (39.5–56.1)	34.7 (27.6–42.7)	40.7 (32.8–49.0)	13.4 (8.4–20.8)	24.2 (17.1–33.1)	19.2 (12.1–29.2)
Nebraska	41.4 (34.1–49.0)	36.8 (30.1–44.0)	37.0 (30.5–43.9)	44.4 (37.0–52.1)	44.0 (37.0–51.3)	41.5 (34.4–48.9)	14.2 (9.5–20.8)	19.2 (13.3–26.8)	21.5 (15.3–29.4)
Nevada	45.7 (37.5–54.3)	33.2 (25.3–42.2)	40.3 (31.9–49.3)	37.9 (30.0–46.5)	45.2 (36.0–54.7)	41.5 (32.9–50.6)	16.3 (11.3–23.1)	21.6 (15.4–29.5)	18.2 (13.0–24.9)
New Hampshire	58.3 (50.5–65.6)	63.8 (56.1–70.9)	65.8 (58.5–72.4)	30.4 (24.1–37.5)	28.6 (22.0–36.1)	23.2 (18.1–29.1)	11.4 (6.5–19.1)	7.6 (4.5–12.5)	11.0 (7.1–16.9)
New Jersey^h^	46.2 (38.8–53.9)	52.3 (43.7–60.8)	58.4 (50.3–66.0)	38.1 (30.5–46.3)	29.9 (23.2–37.5)	26.5 (20.5–33.4)	15.6 (11.3–21.2)	17.8 (11.1–27.4)	15.2 (9.8–22.8)
New Mexico^i^	33.5 (25.2–43.0)	26.7 (20.7–33.6)	35.7 (29.0–43.1)	39.1 (30.4–48.5)	39.8 (32.3–47.9)	43.4 (36.3–50.9)	27.4 (20.1–36.2)	33.5 (26.4–41.6)	20.8 (15.6–27.2)
New York	51.8 (43.9–59.6)	48.3 (44.5–52.1)	48.3 (40.1–56.6)	31.0 (24.2–38.7)	32.9 (29.3–36.7)	35.6 (27.6–44.6)	17.2 (12.1–23.8)	18.8 (16.0–22.1)	16.0 (11.0–22.8)
North Carolina^j^	46.7 (38.2–55.4)	35.3 (27.2–44.3)	43.8 (35.5–52.3)	32.9 (25.2–41.7)	32.8 (24.3–42.5)	34.0 (26.0–43.0)	20.4 (14.2–28.4)	31.9 (23.1–42.3)	22.3 (15.2–31.5)
North Dakota	37.7 (30.9–45.1)	41.3 (33.5–49.6)	39.0 (31.7–46.8)	47.2 (40.0–54.4)	48.3 (40.1–56.5)	46.2 (38.4–54.1)	15.1 (10.4–21.3)	10.4 (6.3–16.8)	14.9 (9.9–21.9)
Ohio	48.4 (42.2–54.6)	44.7 (38.5–51.1)	49.0 (43.6–54.3)	32.4 (27.0–38.3)	34.3 (28.6–40.6)	31.3 (26.6–36.3)	19.2 (14.8–24.7)	21.0 (16.2–26.6)	19.8 (15.6–24.8)
Oklahoma	26.6 (20.2–34.2)	31.2 (23.4–40.2)	29.0 (22.3–36.7)	47.6 (39.7–55.7)	42.5 (33.6–51.9)	46.0 (38.4–53.9)	25.7 (18.7–34.2)	26.3 (19.1–35.2)	25.0 (18.4–32.9)
Oregon	50.9 (46.1–55.7)	50.7 (45.3–56.0)	45.9 (37.4–54.6)	36.9 (32.3–41.7)	37.0 (31.8–42.5)	38.8 (30.4–47.9)	12.2 (9.4–15.7)	12.3 (9.3–16.1)	15.3 (9.8–23.2)
Pennsylvania^k^	54.9 (46.6–62.9)	44.3 (37.9–50.9)	44.4 (38.4–50.6)	32.4 (25.1–40.6)	39.0 (32.6–45.8)	35.0 (29.3–41.1)	12.8 (8.1–19.6)	16.7 (12.6–21.9)	20.6 (15.5–26.9)
Rhode Island	48.7 (40.5–56.9)	58.2 (49.7–66.2)	57.4 (48.8–65.6)	35.7 (27.8–44.4)	30.6 (23.3–39.0)	26.6 (20.2–34.3)	15.6 (10.5–22.5)	11.2 (7.7–16.0)	16.0 (10.3–23.9)
South Carolina	37.0 (29.5–45.2)	40.6 (33.2–48.5)	39.8 (31.9–48.4)	34.2 (26.8–42.4)	35.7 (28.3–43.8)	32.8 (25.6–40.8)	28.8 (21.4–37.6)	23.7 (17.6–31.1)	27.4 (20.3–35.8)
South Dakota	41.2 (34.7–48.0)	44.6 (36.0–53.4)	37.4 (29.2–46.4)	45.4 (38.5–52.4)	43.4 (34.8–52.3)	47.8 (39.1–56.6)	13.5 (9.1–19.5)	12.1 (7.4–19.1)	14.8 (9.0–23.3)
Tennessee^l^	27.8 (21.4–35.3)	32.7 (28.0–37.9)	43.4 (36.7–50.4)	41.7 (33.6–50.2)	40.0 (34.3–46.0)	34.2 (28.1–40.9)	30.5 (23.7–38.2)	27.3 (22.2–33.1)	22.4 (17.2–28.5)
Texas	30.7 (24.0–38.3)	26.4 (19.5–34.7)	35.3 (27.3–44.2)	38.2 (30.4–46.7)	40.6 (32.2–49.5)	34.4 (26.5–43.3)	31.1 (23.5–40.0)	33.0 (24.7–42.7)	30.3 (21.6–40.6)
Utah	32.2 (26.7–38.2)	32.9 (26.2–40.5)	35.3 (28.7–42.5)	46.4 (40.1–52.9)	44.0 (36.5–51.8)	44.2 (36.8–51.9)	21.4 (16.8–26.9)	23.1 (16.8–30.8)	20.5 (14.4–28.3)
Vermont	59.3 (51.5–66.6)	56.9 (48.4–65.0)	59.3 (51.1–67.0)	31.7 (24.8–39.5)	34.1 (26.4–42.6)	33.0 (25.6–41.3)	9.0 (5.2–15.2)	9.1 (6.0–13.5)	7.7 (4.6–12.7)
Virginia^m^	54.4 (45.8–62.7)	43.2 (35.7–50.9)	45.1 (37.3–53.1)	32.2 (24.4–41.1)	38.2 (30.8–46.2)	32.7 (25.9–40.3)	13.4 (8.7–20.2)	18.6 (12.7–26.4)	22.2 (15.5–30.8)
Washington	54.0 (46.3–61.4)	48.1 (39.7–56.7)	53.1 (44.7–61.4)	32.8 (26.3–39.9)	33.7 (26.3–41.9)	34.6 (27.1–43.0)	13.3 (8.6–20.0)	18.2 (12.6–25.6)	12.3 (8.6–17.1)
West Virginia^n^	35.3 (27.9–43.5)	25.4 (19.5–32.3)	37.3 (29.6–45.6)	42.5 (34.7–50.7)	36.0 (28.7–44.0)	43.0 (35.1–51.3)	22.2 (16.7–28.8)	38.6 (30.6–47.3)	19.7 (14.8–25.8)
Wisconsin	48.6 (43.3–53.9)	45.5 (37.2–54.0)	46.1 (40.0–52.4)	38.9 (33.8–44.2)	38.9 (30.8–47.7)	40.0 (33.9–46.4)	12.5 (9.5–16.4)	15.6 (9.6–24.3)	13.9 (9.7–19.4)
Wyoming	34.6 (27.1–42.9)	34.7 (28.5–41.5)	33.0 (27.2–39.4)	52.2 (44.0–60.3)	44.7 (38.0–51.6)	50.5 (43.8–57.2)	13.2 (9.3–18.4)	20.6 (15.2–27.3)	16.5 (11.7–22.8)

^a^ Significant nonlinear trend for ≥4 times per week (*P* = .01).

^b^ Significant linear trend for ≥4 times per week (*P* = .05).

^c^ Significant linear trend for ≥4 times per week (*P* = .03).

^d^ Significant nonlinear trend for 1–3 times per week (*P* < .001).

^e^ Significant linear trend for ≥4 times per week (*P* = .05).

^f^ Significant nonlinear trend for 1–3 times per week (*P* = .02).

^g^ Significant linear trend for ≥4 times per week (*P* = .04).

^h^ Significant linear trend for 1–3 times per week (*P* = .02).

^i^ Significant nonlinear trend for ≥4 times per week (*P* = .02).

^j^ Significant nonlinear trend for ≥4 times per week (*P* = .03).

^k^ Significant linear trend for ≥4 times per week (*P* = .03).

^l^ Significant linear trend for 1–3 times per week (*P* = .01) and ≥4 times per week (*P* = .01).

^m^ Significant linear trend for ≥4 times per week (*P* = .05).

^n^ Significant nonlinear trend for ≥4 times per week (*P* < .001).
